# Use of poppers (nitrite inhalants) among young men who have sex with men with HIV: A clinic-based qualitative study

**DOI:** 10.1186/s12889-024-19284-1

**Published:** 2024-06-29

**Authors:** Nicole Pepper, María Luisa Zúñiga, Heather L. Corliss

**Affiliations:** 1 University of California San Diego, Pediatrics, San Diego, CA USA; 2 School of Social Work, San Diego State University, San Diego, CA USA; 3 School of Public Health, San Diego State University, San Diego, CA USA

**Keywords:** HIV, YMSM, Poppers, Nitrite inhalants, Substance use, Chemsex

## Abstract

Nitrite inhalants (poppers) are associated with HIV transmission and commonly used among young men who have sex with men (YMSM), a group at increased risk for HIV. Significant research gaps exist in understanding the context in which YMSM use poppers. Qualitative interviews were conducted with 15 YMSM (22–31 years) with HIV to better understand the context in which poppers are used and their impacts on HIV care outcomes, such as care retention and antiretroviral adherence. The Social Ecological Model was applied to understand intrapersonal, interpersonal, community, and system level influences on popper use. Factors influencing popper use included: ubiquity of popper use in sexual settings, introduction to poppers by casual sexual partners, patient-HIV provider communication surrounding poppers, neighborhood, substance use and HIV care systems, and the legal status of poppers. Implications for clinical care, public health, policy, and future research are discussed.

Young men who have sex with men (YMSM) experience elevated rates of substance use compared to their heterosexual peers [1–4] and in 2020, 20% of new HIV diagnoses in the United States (U.S.) were among young people aged 13–24, with black and Latino YMSM at greatest risk [5]. Alarmingly, youth are the least likely of any age group to be aware of their HIV status, retained in care and have an undetectable HIV viral load [5]. The science is clear: individuals who receive timely HIV diagnosis, are retained in HIV medical care and achieve sustained viral suppression on antiretroviral medication do not transmit HIV [6]. Given that substance use negatively impacts each stage of the HIV care continuum (diagnosis, entry into care, treatment initiation, and viral suppression), it is essential that substance use is assessed and addressed in vulnerable populations in order to achieve positive health outcomes and reduce HIV transmission [7].

Nitrite inhalants (poppers) are commonly used by men who have sex with men (MSM) [8, 9] and are associated with HIV acquisition [10]—likely due to their association with anal intercourse between individuals of mixed HIV status [11] and condomless sex [12]. Poppers are potent rapid-onset short-acting vasodilators which produce a head rush and are often used during sexual encounters to enhance pleasure and facilitate intercourse by relaxing smooth muscle tissue. They are legal to purchase under the guise of commercial use (e.g., cleaners and odorizers), are widely available online and at adult bookstores and have potential for abuse. Popper use is concentrated in the MSM community; representative U.S. data from 2015 to 2017 indicated that over one-third of men who identified as gay and approximately 11% of men who identified as bisexual had used poppers in their lifetime compared with less than 4% of men who identified as heterosexual [9]. Among MSM with HIV who met criteria for drug abuse, 56.6% were using inhalants [13]. Popper use is often reported broadly as ‘inhalant use’ in representative data and available literature on popper use is often focused specifically on MSM populations. Available literature comes predominantly from the 1980s through early 2000s [14–21], posing challenges to understanding the current potential scope of popper abuse.

Motivation for research involving poppers during the early HIV epidemic stemmed from the concern that popper use was causally associated with HIV-acquisition and/or the development of AIDS [14, 15, 17, 22–25], followed by research establishing the correlation between popper use and HIV via associated risk factors (e.g., condomless sex, anal intercourse between individuals of mixed or unknown HIV status) [10, 11]. Factors that may have contributed to a decline in poppers-related research include the legal status of poppers, the perception that they are not a drug of abuse and a lack of prioritization when compared to other harmful substances (e.g., opiates, methamphetamine). Yet poppers are well-established to be correlated with behaviors that place individuals at increased risk of HIV acquisition and have the potential to impact the health and wellbeing of YMSM, who are often less likely to access health care and have their health care needs met [26]. Our review of the literature highlights significant remaining gaps in our understanding of the implications of popper use on HIV risk and outcomes, specifically for YMSM.

In particular, information is lacking on the individual and social experience of young people with HIV who use poppers and whether poppers influence HIV care outcomes (such as viral suppression). The limited availability of current data underscores the rationale for our current study, indicates that poppers are often overlooked, minimized or under assessed by researchers and clinicians as drugs of abuse, and little is understood about social and environmental contexts for use. Even medical providers with experience providing HIV and addiction care to MSM may not be aware of the risks of popper use [27]. Speculation early in the epidemic that popper use caused AIDS or Kaposi sarcoma has been disproven [28, 29], but gaps remain in our knowledge of their mechanism for HIV risk. There is some evidence that popper use among persons living with HIV is associated with immunosuppression [30, 31] and elevated cancer risk [32], but a richer understanding of the context for behavioral risks associated with popper use is needed and represents an unmet opportunity to reduce new HIV transmissions. Further, the impact of popper use on HIV care engagement (e.g., attending medical visits) and treatment adherence is unknown.

Qualitative research on poppers could elucidate these research gaps. A search of the literature identified only two qualitative studies [33, 34] conducted in the 1990s which do not focus on implications of popper use for individuals with HIV. Researchers have identified the need for qualitative research to better understand quantitative findings regarding the influence of popper use on sexual risk behaviors (e.g., condom usage) among MSM [35]. To address this gap in knowledge, the aims of the current qualitative study are to: (1) contextualize the experiences of popper use among YMSM with HIV, particularly as they relate to HIV care and treatment and (2) describe perceptions of how individual, social and environmental factors impact YMSM’s ability to stay healthy, manage HIV and influence harms related to popper use.

To best address study aims, the Social Ecological Model (SEM) was applied [36, 37] (Fig. 1). SEM provides a rich context for understanding substance use, associated risks and protective factors which acknowledge the complex interplay of micro, mezzo and macro factors influencing substance use, sexual practices and health behaviors. An important feature of the SEM is that levels are nested and interconnected rather than mutually exclusive. Much of the existing literature on popper use focuses on individual factors, placing the burden on individuals to address health disparities—neglecting the context in which individuals are situated, and the influence of relationships, community and systems on health and wellbeing. YMSM with and at-risk for HIV and substance abuse represent a group in need of tailored interventions at the individual, community and system level to promote their health and wellbeing, as well as support optimal adherence to antiretroviral treatment to reduce HIV transmission in the community. These findings may be useful for clinical, public health, and policy efforts supporting YMSM [38–40].

**Fig. 1:**
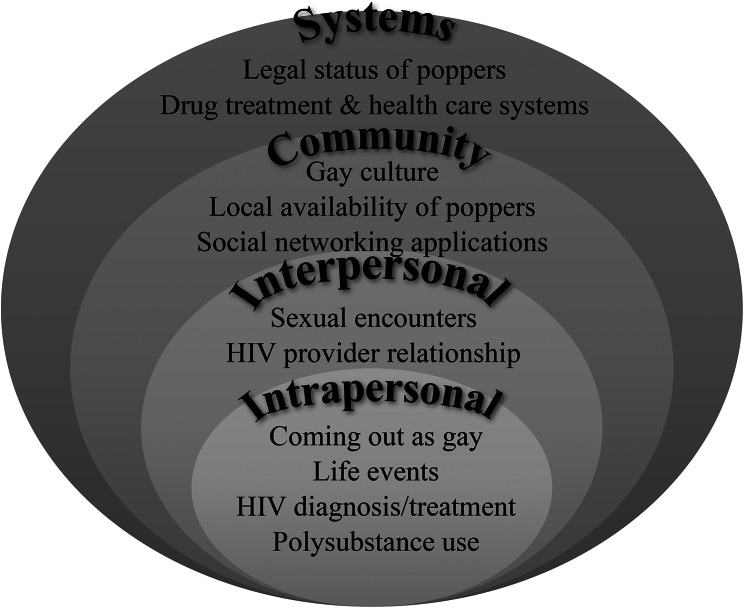
Selected examples illustrating influences on popper use among young men who have sex with men (YMSM) with HIV contextualized in the Social Ecological Model

## Methods

From January 2020 to February 2021, in-depth, semi-structured interviews were conducted using an interview guide with open-ended questions and probes designed to explore individual, social and environmental contexts of popper use among YMSM with HIV (Table 1; interview guide available upon request). Inquiry was guided by four levels of the Social Ecological Model: *intrapersonal*, *interpersonal*, *community*, *systems*. Interviews lasted approximately 60 min and were audio recorded. Due to the COVID-19 pandemic, most interviews were conducted via HIPAA-compliant Zoom for Healthcare. Participants were compensated with a $30 gift card for their time. This study was approved by the University of California San Diego Human Research Protection Program. All individuals provided written informed consent, including consent for audio recording.

**Table 1:** Sample interview guide questions organized by the Social Ecological Model to describe the context of popper use among young men who have sex with men (YMSM) with HIV

SEM Level	Interview Topics	Sample Questions
*Intrapersonal*	Popper initiation Polysubstance use Effects HIV health outcomes	Can you tell me about the first time you tried poppers? I’m wondering if you ever use poppers with other substances. Oftentimes people report both good and sometimes bad experiences while using drugs. What are the positive things that you experience using poppers? How about any negative experiences or side effects? Sometimes people report that using substances impacts their health (for example, taking medications on time) or taking sexual risks when they’re high. How has this been for you with popper use?
*Interpersonal*	Popper use during sexual encounters Medical provider relationship	In what situations do you typically use poppers? Can you tell me about your use of poppers when you’re having sex? What conversations has your medical provider had with you about popper use?
*Community*	Availability of poppers Social networks	Can you tell me about how you usually get poppers? Who else do you know, like friends, partners or acquaintances that uses poppers?
*Systems*	Cost Legal status of poppers	About how much do you usually pay for poppers? Are poppers legal to purchase and use?

A brief demographic survey which included demographic information and basic HIV outcomes (e.g., last HIV care visit, use of antiretroviral treatment and HIV viral load) and a substance use history assessment were completed prior to qualitative interviews. The substance use history assessed lifetime use, age of first use, most recent use, frequency of use, amount used, mode of administration, and concurrent use with poppers for the following substances (in addition to poppers): alcohol, cannabis (including synthetic), cocaine/crack, methamphetamine, opiates (heroin, fentanyl, prescription medications), MDMA/ecstasy, lysergic acid diethylamide (LSD)/acid, other hallucinogens (e.g., psilocybin, mescaline, ayahuasca, N,N-dimethyltryptamine/DMT), non-popper inhalants (e.g., whippets, spray paint, glue), prescription stimulant medications (e.g., Adderall), erectile dysfunction medications (e.g., Viagra), ‘club drugs’ (e.g., ketamine, gamma hydroxybutyrate/GHB and gamma-butyrolactone/GLB), steroids, and other. For prescription medications, participants were asked if the medications were prescribed to them and used as prescribed.

### Participants

Participants were recruited via flyers which were posted in the largest HIV clinic in San Diego, California, which is centrally located in a historically LGBT + affirming, ethnically and racially-diverse neighborhood. In order to expand recruitment reach and diversity of participant experiences and perspectives, flyers were also shared with an HIV clinic specializing in serving young adults and a substance use treatment program tailored to the LGBT + community and individuals living with HIV. Finally, individual outreach via flyers was conducted with HIV medical providers and case managers to inform staff about the study and encourage staff to share the opportunity with their patients. Eligible participants were between the ages of 18–30 years old, HIV-positive, identified as a male who had sex with other men in their lifetime, used poppers in the past six months, were able to provide informed consent, and able to read and speak English. Exclusion criteria included cognitive impairment or currently experiencing symptoms of serious mental illness. If a participant presented with signs or symptoms of impairment or serious mental illness (e.g., confusion, psychosis), established protocol was for the interviewer (a licensed clinical social worker with experience working with youth with co-occurring disorders) to provide clinical assessment and, if needed, referral to emergent services. Additional protocol included requesting to reschedule the interview if the participant presented with acute substance intoxication. No individuals required assessment, referral or study exclusion based on cognitive impairment, serious mental illness or acute intoxication.

### Analysis

Interviews were audio recorded and transcribed verbatim. Personally-identifiable information was not transcribed and interviews were identified only with a unique identifier. Qualitative software (NVivo) was used to conduct analysis. We applied a phenomenological approach [41, 42] to explore and describe the lived experiences of YMSM with HIV who had used poppers. As summarized by Davidsen [36], a phenomenological approach to data analysis follows an iterative process of thoroughly reviewing interview transcripts to identify and interpret the different ways in which participants understand their world and their behaviors [37]. Based on a thorough review of transcripts, the first author identified and discussed potential codes with the research team with the goal of capturing and differentiating meaning across themes. The first author then applied the codes, iteratively refining them in order to organize and articulate rich descriptions of the participants’ perspectives across themes, with continuous discussion of coding interpretation among all three researchers. Sample size was determined by a priori thematic saturation [43]. Researchers determined a high degree of saturation was reached when each level of the SEM was thoroughly explored, as evidenced by ample data reflecting both diverse experiences and similar perspectives to illustrate each level, and when interviews were not producing new information. Thematic saturation was reached with 15 participants. Transcribed interviews were thoroughly reviewed and coded by theme within the four levels of the SEM by the first author. Analysis was also considered within the context of existing literature about popper use among YMSM. Final analysis was conducted with discussion and consensus between researchers. Compelling quotes which illustrate themes were selected from the coded data.

## Results

Participant demographics are shown in Table 2. All but one participant identified as gay and one participant identified as pansexual. Participants predominantly identified as Hispanic/Latino, had completed high school, were unemployed and/or students, and living below the Federal Poverty Level. Average age was 26 (range 22–31 years, SD = 3). 80% of the sample considered themselves stably housed, but a significant number of these participants lived with family or friends or were in time-limited residential treatment or sober living. All participants were engaged in HIV care (attended a visit with their HIV provider within the past six months) and virally suppressed. The average age of first drug use (including cannabis) was 15 (range 8–28 years, SD = 5) and average age of first popper use was 20 (range 12–28, SD = 4). Median days since last popper use was 11 (range 0-178, mean = 45, SD = 64). Pseudonyms have been used to protect privacy.

**Table 2:** Characteristics of young men who have sex with men (YMSM) with HIV participating in a clinic-based qualitative study on popper use

Variable	*N* = 15
**Years of age, mean ± SD (range)**	26 ± 3 (22–31)
**Hispanic/Latino descent, n(%)**	
*Yes*	11* (73.3)
*No*	4 (26.7)
**Which group best describes you?, n(%)**	
*Caucasian*	14 (93.3)
*Other*^*✝*^	1 (6.7)
**Sexual Orientation**	
*Gay*	14 (93.3)
*Pansexual*	1 (6.7)
**Education (highest completed), n(%)**	
*Did not complete high school*	5 (33.3)
*High school completion*	3 (20)
*Some college*	5 (33.3)
*Bachelors Degree*	2 (13.3)
**Employment Status**	
*Employed full-time*	5 (33.3)
*Employed part-time*	2 (13.3)
*Unemployed*	5 (33.3)
*Student*	3 (20)
**Housing**	
*Stable*	12 (80)
*Unstable*	2 (13.3)
*Homeless*	1 (6.7)
**Income**	
*Below Federal Poverty Level*	8 (53.3)
*Above Federal Poverty Level*	7 (46.7)
**HIV Outcomes**	
*Years since HIV diagnosis, mean± (range)*	5 ± 3 (0–10)
*Medical visit in the past 6 months*	15 (100)
*On antiretroviral therapy*	15 (100)
*Virally suppressed*	15 (100)

SD = Standard Deviation

* 10 participants identified as Mexican and 1 as Central or Southern American

✝ Middle Eastern

### Patterns of popper use

All participants reported primarily inhaling poppers nasally directly from the bottle. A few participants described oral inhalation via bagging or huffing, typically either because of tolerance or skin irritation around their nose from repeated nasal inhalation. Less common routes of administration included crushing and snorting poppers after drying the liquid, smoking the liquid from a bong, mixing with alcoholic beverages, and accidental ingestion. Participants reported paying between $10 and $30 dollars per 10mL bottle. Most participants reported using “spray poppers” (aerosol solvents) and noted these were very common in their community. These inhalants were described as more intense than poppers, and in some cases, a progression from conventional poppers to aerosol solvents (sometimes used simultaneously).

Frequency and amount of popper use varied in this sample. At the time of the interview, some participants disclosed that they were in substance use recovery and among them were individuals who reported not using poppers currently. However, participants were asked to categorize how often they typically used poppers during periods of use in the past six months. Nearly all participants reported at least monthly use and more than half reported using poppers at least weekly. Four participants used daily or almost daily. Use varied from periods of total abstinence to moderate to heavy use. Periods of abstinence were attributed to intentional efforts to reduce substance use, participation in substance use recovery services or being abstinent from sex. Moderate (occasional) use was attributed to use as a sexual tool based on sexual positioning (e.g., receptive versus insertive) and partner anatomy and in the absence of polysubstance use. Periods in which heavy use occurred included initiation of popper use, HIV diagnosis, and frequent sexual activity or use of other drugs (“being in my addiction”). Some participants described initial fear of popper use followed by increasing comfort level with continued use. Typical use ranged from one to four sniffs to 15–20 sniffs in a three to four hour period. The high was typically perceived to have lasted a short time—between 10 seconds to a minute.

Although poppers were used almost exclusively during sexual encounters with partners, a number of individuals used poppers by themselves, primarily for masturbation and to a lesser degree “for fun” and to alleviate boredom and stress. Less common settings for popper use included clubs, parties and for kissing: *“Personally, I enjoy poppers when it’s not in a sexual interaction. Once I tried it on the dance floor and I tend to enjoy it the most either when I’m making out or dancing (“Amir,” Middle Eastern, 31 years old).”*

### Benefits and side effects

Participants were mostly aligned about the benefits, side effects and risks of popper use. The primary reported benefits were intensifying sexual experiences, a head and/or body “rush”, and a relaxing of smooth muscle tissue which facilitated anal and oral sex and reduced pain for the receptive partner. Other benefits included stress reduction, relaxation and enhanced intimacy. *“The feeling that I got was fast blood flow throughout my body and relaxation…The rush that it gives, the enhanced sensations, kind of gives me a calmness (“Amir,” Middle Eastern, 31 years old).” “It’s like a head rush. I guess at a certain point I like the feeling, like, how disoriented—it’s just like, you just got dropped in the middle of the ocean (“Diego,” Latino, 24 years old).”*

Overall, participants perceived poppers to be relatively low risk, sometimes citing their short duration, and participants noted they lacked factual information about poppers and risks of use. *“I’ve never really put thought into [poppers], you know, just because it’s so normalized. But it does leave me questioning. To be honest, I don’t even know what poppers are. I don’t know. This whole time I’ve been putting them in my body and using them…If they’re chemical and they’re like a cleaning kind of way…well that’s about all I know about them. Other than that I don’t know anything (“Diego,” Latino, 24 years old).”*

All participants experienced headache as a common side effect and oftentimes it was the side effects that caused participants to wonder about safety and prompted participants to research poppers. Others reported feeling dizzy, nauseous, lightheaded, dehydrated, and having dry mouth. One participant reported having passed out and experiencing auditory hallucinations while using poppers. *“It just always has the same effect on me. I never really liked it. It just gives me a headache instantly and I ask myself why I did that ‘cause I know how it affects me…some of them have been like where I sniff it and then immediately it’s like a truck hits me…it’s like a 15-pound weight in the front of my skull and it’s like dragging me down everywhere I go. And it’s a splitting headache (“Mateo,” Latino, 27 years old).” “The headache that I experience is not very pleasurable afterwards so I know it’s not a healthy substance (“Amir,” Middle Eastern male, 31 years old).”* Participants reported learning more about poppers online, through friends or sexual partners, from adult bookstore employees, or through popper-specific pornography and Reddit boards. Several participants reported that poppers made it difficult to maintain an erection. Participants expressed mixed opinions regarding whether they developed a tolerance to poppers or experienced a progression of abuse. *“The feeling can be different every time you do it because depending on the poppers, on how you’re feeling–it’s just like every time can be different, but I feel like the rush is always the same. Like with methamphetamine, I feel like I have a tolerance with that, but with poppers, it’s like I don’t have a tolerance, like every time I use it it’s the same, you know (“Miguel,” Latino, 22 years old).”*

## Contextualization of popper use and HIV risk and outcomes in the social ecological model

### Intrapersonal

#### Initiation

For most participants, popper use preceded their HIV diagnosis. Common personal factors influencing popper initiation included general experimentation with substance use and disclosing sexual orientation (“coming out”). Other factors included increased freedom (e.g., moving away from home, getting their own apartment, graduating college) and adverse life events (e.g., personal loss, family difficulties, break-ups). Several participants described the time in their life surrounding popper use as “self-destructive” or a “downward spiral.” *“I was more experimental. I had just joined a dating app, or not even dating, it’s like Grindr. I was just putting myself more out there. I had come out. I was meeting people. It was just, like, a new chapter. Like my whole world was really unfolding cause I’d just graduated college (“Juan,” Latino, 26 years old).”* HIV diagnosis was also identified as a factor that led to popper initiation or increased use. *“Yes, it [popper use] increased a lot [after my HIV diagnosis]. It increased a lot (“John,” Non-Hispanic White, 29 years old).” “I had just gotten diagnosed with HIV…and at that point, I just didn’t care about life anymore so I was willing to try anything (“Joaquín,” Latino, 29 years old).”*

#### Polysubstance use

Polysubstance use with poppers was consistently reported by participants. Table 3 describes lifetime substance use and whether the individual reported having used a substance simultaneously with poppers. Nearly all participants had used methamphetamine with poppers and nearly three quarters (73%) of participants had used poppers with club drugs, like ketamine and gamma-hydroxybutyrate (GHB), and/or with erectile dysfunction medications (e.g., Viagra, Cialis).

**Table 3:** Lifetime substance use (including concurrent use with poppers) among young men who have sex with men (YMSM) with HIV participating in a qualitative interview about the context of popper use

	Lifetime Substance Use *n*(%)	Used with Poppers *n*(%)
*Alcohol*	15 (100)	8 (53)
*Cannabis*	15 (100)	8 (53)
*Cocaine*	9 (60)	3 (20)
*Methamphetamine*	13 (87)	13 (87)
*Heroin*	6 (40)	1 (7)
*Fentanyl*	4 (27)	1 (7)
*MDMA, Ecstasy*	12 (80)	6 (40)
*LSD*	8 (53)	1 (7)
*Hallucinogens*	10 (67)	2 (13)
*Other Inhalants*	10 (67)	3 (20)
*Stimulant Pills*	8 (53)	2 (13)
*Pain Pills*	7 (47)	1 (7)
*Sedative Pills*	10 (67)	3 (20)
*Erectile Dysfunction Medications*	12 (80)	11 (73)
*Club Drugs (e.g. ketamine, GHB)*	12 (80)	11 (73)

Participants in this study resoundingly shared that popper use was more likely when other substances were being used. *“Your body turns into just some chemical power plant. There’s meth, there’s poppers, there’s Viagra, you know? There’s alcohol. It’s just a wreck waiting to happen. Usually with meth use it [popper use] would increase a lot (“John,” Non-Hispanic White, 29 years old).” “When I started using meth about three years ago, where I would just do them [poppers] every time that I was high on meth, I’d just be on poppers all the time…I wouldn’t necessarily go out of my way to get ‘em, you know what I mean?…I would do [ketamine] every day, along with GHB, meth and poppers. It was all part of my combo—my routine at that moment in my life (“Daniel,” Latino, 30 years old).” “It [poppers] adds another level to it [co-use of GHB, methamphetamine and Viagra], so it makes it a little bit more fun and intense and it makes it last a little bit longer. It just makes it stronger, to be honest, whatever you’re feeling (“Miguel,” Latino, 22 years old).”*

Some mentioned co-use of other drugs to mask the unpleasant side effects of popper use, like headaches. *“If I’m sober, I don’t really want to [use poppers], ‘cause I just don’t really like how they make me feel…I mostly only use them when I’m on other substances (“Devon,” Non-Hispanic White, 23 years old).” “Pretty much they [methamphetamine and poppers] always went together. I remember a bunch of occasions in, like, sober sex, when you know, they usually give you a headache, so I wouldn’t usually use them outside of using meth (“John,” Non-Hispanic White, 29 years old).”* Poppers were also used in the context of polysubstance use in an effort to restore an erection when methamphetamine use resulted in impotence. *“You know, where the methamphetamine kicked in and you got ‘T-dick’ or whatever, and it’s like well there’s really nothing to do so you’ll just use poppers, you know, to help rebuild your erection (“Luis,” Latino, 24 years old).”* It was very common to report concurrent use of poppers and unprescribed erectile dysfunction medications and some participants were aware of the contraindication. *“There’s usually, like, Viagra involved. Meth, Viagra, poppers, other inhalants. I am aware that it’s a high risk for like heart attack or, you know, cardio issues (“John,” Non-Hispanic White, 29 years old).”*

It was common for participants to minimize popper use in the context of other drug use. Not all participants abstained from poppers during periods of sobriety, although many did, and several identified popper use as a risk factor for relapse with other drugs. *“I never saw it [popper use] as, like, really a problem, just ‘cause, I was on other drugs so I never really…thought about it in that way, but I feel like they go hand in hand with drug use…but I never really thought that, like, poppers were an issue (“Nic,” Latino, 29 years old).” “I’ve literally relapsed before over, you know, I went and I hooked up with someone and they had poppers…led me to a few days later doing meth (“John,” Non-Hispanic White, 29 years old).”*

#### Perceived risk

Most participants shared a low perception of risk and expressed ambivalence about use; individual factors noted by participants to influence risk perception included lack of knowledge about poppers, the short duration of effects, perceived lack of overdose risk, and the perception that poppers weren’t addictive or weren’t “hard drugs.” “*To me they’re not really a drug. I mean, they are definitely a drug, but they don’t last long, you know?…I use them…in sexual situations. I use them as a tool, ‘cause sometimes they are necessary…I don’t include poppers in my sobriety. I don’t think that they should. They don’t show up on a drug test and, like I said, I mean, I don’t use them outside of sexual contact and context…I don’t think that poppers really affect my judgment…it’s not like a hard drug to me…to me they’re not addictive. Like I definitely want to use them less. I do know they have a pretty big effect on…my health in general… so I will probably start using them less. (“Ian,” Non-Hispanic White, 27 years old).”* The interconnectedness of SEM levels is exemplified in this quote. The combined effect of individual (e.g., duration of effects), interpersonal (contextual use as a sexual tool versus a drug of abuse) and system level influences (not showing up on a drug test) combine to shape perception that risks associated with use are low and influencing behavior (continued use during sexual encounters).

Despite the perception of relative low risk associated with popper use, participants described heavy use and a progressive relationship with poppers. *“I had an issue with inhalants and I needed to stay away from them, ‘cause it was progressive (“John,” Non-Hispanic White, 29 years old).” “If they [poppers] were there, I was gonna use them and I was gonna use them a lot. Until, like, my nose hurts, I couldn’t breathe anymore. And then I would still try to use them with my mouth…I would hit that point a lot (“Daniel,” Latino, 30 years old).”*

#### Popper use and HIV risk and treatment adherence

Few participants had considered a direct connection between popper use and HIV and did not feel that popper use impacted their ability to be adherent to antiretroviral treatment or remain engaged in HIV care, again citing the short duration of their effects. One participant alone expressed concern that poppers might decrease the effectiveness of his HIV medication. *“They may, you know, decrease the effectiveness of my medication or whatever. I do realize that…, but as far as them causing me to not take the medication or, like, condom use, I don’t think that they have an effect on that (“Ian,” Non-Hispanic White, 27 years old).”* Participants attributed poor medication adherence and care retention to other drugs they often used with poppers, primarily methamphetamine. On the other hand, some participants were intentional about taking their HIV medication and prioritizing their health during periods of drug use. *“When I use poppers or when I use any kind of substance, I’m responsible. I take my meds, I make sure after I’m done I go get tested [for sexually transmitted infections]…even when I’m using drugs or poppers, I take my meds every day (“Luis,” Latino, 24 years old).”*

### Interpersonal

Interpersonal influences, primarily through sexual partners, were a major factor contributing to popper use. Interpersonal influences on popper use were identified as contributing to HIV risk in several ways: initiation of popper use with sexual partners, increased sexual arousal and prolonged sexual encounters, disorientation, inhibiting conversations about condom use or HIV status, multiple partners and group sex, polysubstance use with drugs associated with sex (e.g. methamphetamine, GHB, Viagra), popper used based on the anatomy of the insertive partner (potentially leading to increased tearing or bleeding), masking pain, rougher sex, and sexual assault. *“I feel like poppers are related to HIV because sometimes it takes out that conscience that you have in your head when you’re with, like, a sexual partner in the room, and once you take that whiff, it’s like a drug where like all bets are off…so all you’re there for is basically sex and if that’s on your mind once you whiff poppers, there’s not typically a conversation about HIV or anything. It just throws that out the window (“Luis,” Latino, 24 years old).”*

#### Popper use in the context of sexual relationships

All participants had used poppers during a sexual encounter, most almost exclusively in this context. Increased sexual activity heavily influenced popper initiation and frequency of use. *“I was having a lot a lot of sex…my whole life revolved around sex. I finally had freedom, I had a place [to live], it was what I did for work, it was what I did for everything…So it was like poppers poppers poppers (“Daniel,” Latino, 30 years old).”* Some participants defined frequency of popper use in relation to sexual activity (e.g., using poppers “like 90%” or “70%” of sexual encounters).*“The more sex I had, the more I would use poppers, I guess, cause they were always around…so I would always just, just use poppers cause I was having sex (“Diego,” Latino, 24 years old).”*

Perceived benefits of popper use during sex included intensifying pleasure, facilitating anal and oral sex by relaxing smooth muscle tissue and reducing pain and bleeding. Some participants described poppers not as a drug, but as a “tool” or “accessory” for sex. Easing tension or anxiety, emotional numbing, and artificial intimacy were also cited as reasons for using poppers in sexual settings. *“All my muscles got relaxed for a second and then it was easier for the guy to, you know, for us to have sex. It helps you loosen up (“Carlos,” Latino, 22 years old).” “When you use poppers, it relaxes your sphincter muscles. That way, it makes it easier for them to be able to penetrate you and not be as painful (“Miguel,” Latino, 22 years old).” “There was once when I didn’t use them [poppers] and they [my partner] were very big and I did bleed (“Juan,” Latino, 26 years old).”“It’s just, like, so it wouldn’t hurt I guess…it all depends on size, you know? ‘Cause…if it’s big, it’s gonna hurt and I just didn’t want to feel it (“Sam,” Latino, 22 years old).” “Intimacy, I guess. [Poppers] makes it…feel real, not just like a hook-up (“Joaquίn,” Latino, 29 years old).” “If I just really don’t want to do it [have sex], it helps me not be so tense (“Carlos,” Latino, 22 years old).”*

The majority of participants used poppers for the first time during a sexual encounter with a casual partner and some had never heard of poppers prior to the experience. Poppers were usually supplied at the suggestion of their partner as a way to make anal sex more comfortable. *“The first time I tried poppers it was kind of like a suggestion from the man I was having sexual intercourse with. It would relax my body…They suggested, they offered. They just said, ‘Take one sniff, you’ll feel relaxed.’ (“Luis,” Latino, 24 years old).”* Participants discussed popper use almost exclusively in the context of “hook-ups” or casual partners (primarily met via applications such as Grindr and Adam4Adam) and few participants reported using with primary partners.

There was a perception that poppers were used primarily by the receptive partner—both both because they relaxed muscle tissue and made it easier and less painful for receptive sex and also because insertive partners experienced difficulty maintaining an erection with popper use. *“I would try to use more poppers to continue my erection…to pretty much continue staying in that elevated state so I can maintain the erection, but that only lasted for so long (“Nic,” Latino, 29 years old).”* However all but one participant reported being versatile (receptive and insertive) and having used poppers regardless of sexual position. Overall participants reported that in their experience, both partners used poppers during a sexual encounter. Despite this, the idea that poppers were used primarily for receptive partners persisted. *“Usually both [use poppers]. Like if my partner is using it, then I’ll use it or he’ll ask me if I want to use it…A lot of my friends, they mostly use it when they bottom (“Diego,” Latino, 24 years old).” “I would say that’s not true [that poppers are used only for the receptive partner], because you still get the rush. But it’s usually mostly for bottoms, because it’s, like, I guess, more painful to be a bottom, but I mean, it’s as pleasurable for the top too (“Miguel,” Latino, 22 years old).” “I typically bottom, but I’ve in recent times topped more and, I mean, I still use them in those situations…but it makes it difficult if you are topping to use them. They are kind of an erection killer (“Ian,” Non-Hispanic White, 27 years old).”*

Risk for assault (both sexual and physical) and theft were identified as safety concerns related to popper use. Two participants had experiences during which popper use was not consensual. *“You can do too much and, like, pass out and that can be unsafe for anyone because you don’t know where you’re at or who you’re with…and they could fully take advantage of you either sexually or just rob you (“Luis,” Latino, 24 years old).” “It affects my awareness, you know? ‘Cause you get that head rush, you know, you’re kind of, like, dizzy, spinny and there are some guys out there that kind of took advantage of me in some situations, you know?…I became less aware and conscious of what was really going on around me, which is dangerous (“John,” Non-Hispanic White, 29 years old).”*

Few participants considered a direct risk between popper use and HIV, but participants did speak to popper use enabling sexual risk taking. Few participants felt that popper use impacted condom use, because few participants reported using condoms regardless of substance use. *“As for taking sexual risks, I have taken sexual risks because of poppers (“Juan,” Latino, 26 years old).” “I’ve had a couple friends that have been raped or, like you know, influenced, probably persuaded into having sex and, you know, poppers are always part of it. So it’s, you know, I could see maybe a link [to HIV] somewhere (“Diego,” Latino, 24 years old).” “I go back to awareness, you know? What’s going on lowers inhibitions…I’m in like some head high…I am literally mentally distracted…I know for a fact, that there’s a correlation between [poppers] and…less protected sex and getting HIV. Like the correlation is so strong, it’s crazy (“John,” Non-Hispanic White, 29 years old).” “I really don’t think that they [poppers] have had an effect on me not using condoms (“Ian,” Non-Hispanic White, 22 years old).” “The euphoria, the disorientation, you know, it affected my judgment a 100%. So I can easily see myself, you know, doing poppers and not being as cautious to use a condom for sure (“John,” Non-Hispanic White, 29 years old).”*

Participants articulated protective factors they associated with popper use (e.g., reducing other substance use, supporting medication adherence and reducing bleeding during sex). *“It [poppers] actually helps, because I feel like I don’t need to smoke [meth] to, you know, not hurt [during sex] (“Joaquίn,” Latino, 29 years old).”* Several participants strengthened their commitment to medication adherence during periods of substance use as a way to reduce risk of HIV transmission to their partners. *“Knowing that I was going to use poppers again kind of made me want to…make sure that I took my medication, you know? Just in case that accident happened again where I forgot to put on a condom or the person did or whatever…that I was as close to not being able to infect the person as possible, you know? So the poppers kind of got me into taking my medication more frequently and to make sure I was taking it (“Leo,” Latino, 24 years old).”*

#### Provider relationships

Participants in this study reported positive and supportive relationships with their HIV providers and welcomed discussions about substance use. *“[When my doctor asks about substance use] I feel like they care and I see that they care and I’m happy that they do care. I do not mind, you know, I like to hear it ‘cause then it shows that they still care (“Carlos,” Latino, 22 years old).”* Many felt that HIV providers were more knowledgeable and nonjudgmental about substance use than general practitioners. Having a provider who was a member of the gay community was also cited as a factor increasing trust and comfort level. Interestingly, although nearly all participants discussed substance use in general with their provider, and felt comfortable doing so, none had discussed popper use. Participants reported their providers had never asked about poppers. *“We haven’t really talked about poppers. I don’t think she’s asked me. We’ve talked about meth. She’s given me resources; she set up an appointment with their drug counseling. I’m pretty comfortable with her. She’s pretty open-minded. I mean, she’s an HIV specialist (“Ian,” Non-Hispanic White, 27 years old).”* For some, providers not asking about poppers reinforced the perception that poppers were not dangerous or addictive and others attributed lack of assessment of popper use to prioritizing other substance use (e.g., methamphetamine) or focusing on HIV outcomes. *“Since they don’t make a big deal about it at the doctor—all they care about is my T cell count and all that, you know? They don’t really care about what drug I’m using (“Miguel,” Latino, 22 years old).”*

### Community

Community factors influencing popper use included: local availability of poppers, neighborhood, peer group, perceived norms around condom and substance use, gay culture, use of applications like Grindr and Adam4Adam, and community awareness and risk perception.

#### Local availability

Poppers were widely available in the community. *“It’s something that you can get practically around the city anywhere (“Diego,” Latino, 24 years old)?”* The most common means of acquiring poppers was through sex partners and purchasing at adult bookstores. *“I wouldn’t buy ‘em, they would just be there—like, my sex partner would have them. I only used poppers when it was just around, if they offered it (“Sam,” Latino, 22 years old).”* Participants also purchased poppers at liquor stores and online (e.g., eBay, Amazon, Craigslist). Most participants resided in Central San Diego, inclusive of Hillcrest which is known as an LGBT + affirming neighborhood. Several participants mentioned Hillcrest specifically in reference to poppers. One participant shared that in his experience, popper use was more common in San Diego than other places he had lived. *“A lot more common than [name of state] for sure…I had never tried it out there at all and then when I got out here [San Diego], it’s everybody is doing it, everybody has it (“Joaquίn,” Latino, 29 years old).”* Peer group was also mentioned as a factor influencing popper initiation. *“I was hanging out with the wrong crowd (“Sam,” Latino, 22 years old).”*

#### Perception of poppers and gay culture

Participants shared a perception that popper and other substance use was common in the local gay community, contributed to HIV risk and that poppers were associated with gay culture and subcultures (e.g., the leather community). *“It was almost like gay culture to have poppers (“Diego,” Latino, 24 years old).” “It’s [use of poppers] built into the gay party scene I guess, the culture…People that use poppers are more likely to have HIV, you know? It’s ‘cause, like, those type of crowd and scene that they’re all into (“Carlos,” Latino, 22 years old).”* Meanwhile, participants shared a sense that awareness about the risks of popper use in the community was low. *“The lack of education, the lack of social awareness. I think people, in my perception, I think that people rationalize it, like ‘Oh I mean, it’s only poppers. It’s just, like—you know—a sex accessory (“John,” Non-Hispanic White, 29 years old).” “A lot of my friends—maybe like 90% of my friend group—is straight, so like, when they heard about it [poppers], they didn’t know what I was talking about. So it’s really limited to, in my world at least, the gay community…I’m not educated enough on [poppers], nor have I done enough research. I think mostly because I just relied on the information that I’ve received from the community (“Juan,” Latino, 26 years old).”*

Participants shared about a normalization of condomless sex and drug use associated with “hook-up culture” and use of social networking applications (most commonly Grindr and Adam4Adam) to meet sexual partners with whom they used poppers. Mention of drug use on these applications was ubiquitous and in some cases the applications even allowed for users to filter results to identify partners who used drugs. Sometimes poppers would be discussed prior to meeting in person. Meeting sexual partners on social networking applications, especially around the time of coming out as gay, was identified as an important part of the context for popper initiation. *“[On social networking applications] a few times I would be like, ‘What are you into?’ And people would say, like ‘Oh poppers, are you okay with poppers?’ It’s very seldom [that we would discuss it] ‘cause it’s such a common thing. So you know, it’s almost, like, so socially normal for, like, everything—or it seems like, a lot like for the gays to be okay with that (“Diego,” Latino, 24 years old).” “It’s pretty common [in the gay community], yeah. I will see on profiles, like ‘Poppers Plus’ or something like that. It’s like a thing to talk about if you’re going to have sex (“Juan,” Latino, 26 years old).”*

### Systems

The primary system level influences on popper use were the legal status of poppers and drug treatment and HIV health care systems.

#### Legal status of poppers

The fact that poppers are legal to purchase was frequently cited as influencing perception of low risk, yet participants were confused about the legal status of poppers. Most were aware that poppers were legal to purchase, but less clear on whether they were legal to possess or use. Poppers were typically displayed near the entrance of the store, in a refrigerated case that was accessed by employees. Participants were aware that there was a specific way to purchase them and that they would be denied if they asked for “poppers,” but were not clear on the rationale. Instead, participants asked for specific brand names (e.g., “Rush” or “Jungle Juice”), cleaners (e.g., video head cleaner) or “nail polish remover.” Participants shared that no identification was requested to enter stores or purchase poppers and that websites where poppers are purchased did not ask for age verification.

"*I don’t think they’re supposed to be used the way that they are used. I don’t know if it’s legal (“Devon,” Non-Hispanic White, 23 years old).” “You want to make sure you ask for the right thing, because if you ask for poppers—they say they don’t have poppers…you have to ask for something like video head cleaner or something like that. I would just ask for the brand sometimes (“Daniel,” Latino, 30 years old).” “So from my understanding, when you go into sex shops, if you say, like, ‘poppers’, they can’t legally sell them to you, I think. You know, which is horrible, because no one is using video head cleaner that’s shopping at a sex shop (“John,” Non-Hispanic White, 29 years old).” “Well because they’re being sold for a purpose other than what you’re using them for and the terminologies or the verbiage that you use would indicate that you know you’re going to use them to get high off them. So in order to, you know, superficially look like you’re buying them for the intended purpose of them, you have to use the language that they use on the bottle (“Mateo,” Latino, 27 years old)."*

#### Systems of care

All participants were engaged in HIV medical care (had attended a medical visit and completed HIV monitoring labs within the past six months) and 87% had at least one substance use treatment episode. Despite heavy popper use, few identified poppers as one of the substances for which they sought treatment. Participants had received substance use assessments (e.g. questionnaires and urine tests) in the context of their medical care, but they did not include poppers and participants pointed out that poppers were not detected on urine toxicologies.

With few exceptions, participants said that popper use was not discussed in their substance use treatment programs and that there was hesitancy to define sobriety as inclusive of abstinence from poppers. Although some participants did not include abstaining from poppers as part of sobriety, others felt that popper use should be better addressed in treatment noting that they led to relapse of other drugs. *“No, I don’t think we ever talk about poppers here [in residential substance use treatment]. I mean, it’s such a gray area because it’s legal. I guess it gets overshadowed. It gets overlooked by a lot of people in recovery are like, ‘Well poppers are okay for me to use,’ you know? And then that becomes like the gateway back again to their active addiction. I know I used poppers before and then I relapsed, like, within the next few days (“Diego,” Latino, 24 years old).” “I feel like sometimes counselors don’t want to tiptoe or, you know, create hard lines in the sand of what’s considered relapse because they don’t want to, you know, scare people off. But, you know, it’s how I define sobriety is, anything that affects me, my body from the neck up, my mind—literally any mind altering substance. But I don’t think it’s talked about. I think education around the effects that poppers and other inhalants can have, you know, on your body, especially like the toxicity of it on your brain and your lungs, I think that would benefit a lot of LGBT rehabs (“John,” Non-Hispanic White, 29 years old).”*

## Discussion

Following we consider our findings illuminating novel and critical aspects of popper use in relation to their ability to inform clinical care, public health strategies, policies, and future research to reduce harms and promote the health of YMSM with and at-risk for HIV.

### HIV care and substance use treatment

Most participants were not well informed about poppers’ potential adverse effects, especially more serious risks, and expressed a desire to be more informed. Adverse effects of poppers range from mild to potentially life-threatening and include contact dermatitis, neurotoxicity, vision problems, methemoglobinemia, and serious hypotension [16, 44]. Ingestion [45] and/or concurrent use with other substances can elevate risk for serious side effects. Most participants had used poppers with Viagra, which is contraindicated [46], and concurrent use represents potential correlates of other risk behaviors like condomless sex, polysubstance use and assault. Viagra also interacts with some antiretroviral medications (protease inhibitors) [47], causing it to be metabolized more slowly. Individuals on protease inhibitors using Viagra and poppers are at potentially increased risk for serious hypotension [47]. Since most participants did not have a prescription for erectile dysfunction medications, medical providers should include screening and counseling about these medications during substance use assessments.

Popper use was not perceived as impacting HIV care engagement or antiretroviral treatment adherence. While participants did not directly attribute HIV risk to popper use, they described popper use as part of a constellation of other sex and drug risk behaviors–offering multiple potential avenues for intervention and education. Several participants indicated that popper (and other drug) use increased after HIV diagnosis; this has been attributed to coping with HIV-related stigma, denial and false information about the morbidity and mortality of HIV [48]. Substance use assessment and counseling may be an important part of supporting YMSM at the point of HIV testing and diagnosis, as a way to promote their health, link to needed support services and reduce risk of transmission.

Screening for popper use should occur as part of HIV primary care and, ideally, in primary care settings serving sexual minorities—especially as an opportunity to provide education on pre-exposure prophylactic treatment (PrEP). Despite trusting and supportive relationships with HIV care providers, including open dialogue about other substance use, participants had not been asked by their providers about popper use. This represents a critical missed opportunity for risk reduction and health education and reinforced participants’ perception of poppers as relatively low-risk substances. Currently, none of the commonly used substance use screening tools recommended for HIV care settings [49] include poppers specifically. Based on our findings, we recommend that HIV care providers ask their patients directly about popper use (and use of nonprescription erectile dysfunction medications) to facilitate a discussion including health education and risk reduction, context for use, and patients’ motivations for and perceived benefits of use. Positive relationships with HIV care providers and existing conversations about substance use are an excellent foundation on which to build in assessment and education about popper use and related sexual behaviors. Assessment of legal substances (e.g., cannabis and alcohol) is also important since the legal status of poppers influences use and risk perception and studies have identified a strong association between cannabis and popper use [9]. Participants in this study had all used cannabis–in most cases prior to popper use.

In this sample of YMSM with HIV, most had received substance use services (e.g., outpatient and/or residential care) and shared positive experiences with treatment. This is encouraging and potentially points to service systems that enable YMSM to connect to treatment and effective referral partnerships between HIV care and substance use systems. One participant specifically mentioned that his HIV provider referred to substance use treatment, which was coordinated within the same health care system. Recovery services that were tailored to the needs of young adults, individuals with HIV and sexual minorities and were centrally located (and/or integrated into their HIV care) in areas frequented by YMSM promoted engagement in substance use services. Participants identified a need for substance use treatment to address poppers specifically and in the context of polysubstance use, as well as for interventions that considered the role sex and popper use played in relapse on other substances (e.g., methamphetamine).

Participants expressed considerable ambivalence about popper use–their risk, context, effects, and connection to HIV. This ambivalence presents a ripe opportunity for interventions aimed at reducing potential harms. Evidence informed interventions for substance abuse, like motivational enhancement approaches and Screening Brief Intervention and Referral to Treatment (SBIRT), are brief interventions that have been successfully implemented in various settings (e.g., primary care, emergent care, substance use treatment) and by various disciplines (e.g., medical providers, social workers, case managers, peers, and alcohol and drug counselors) [50]. While no evidence informed treatments currently exist specifically for popper or inhalant abuse, basic screening, brief intervention (e.g., health education) enhanced by motivational approaches and, when needed, referral to substance use programs could be of great benefit. SBIRT is beneficial as both an intervention and as prevention, since it provides an opportunity for a dialogue between provider and patient and provision of basic educational information.

### Public health

Popper use among YMSM with HIV impacts public health due to the correlation of popper use and HIV transmission. There is a need for enhanced population level screening and dissemination of information related to potential risks, especially given study participants’ perspective that popper use is part of gay culture. Unfortunately, poppers are often absent from validated screening tools or included generally under the category of inhalants. Other inhalants (e.g., spray paint, whippets, glue) have very different contextual risks. In order to effectively address popper use, service systems should also incorporate assessment of gender identity and sexual orientation as part of routine standard care.

Participants perceived that overall community knowledge about potential risks of poppers was lacking. Studies on popper use from 1978 [51] and 1997 [34] similarly found that participants felt ill-informed about side effects, demonstrating that community health education (e.g., public service announcements and campaigns) is long overdue. Specific public health efforts are needed to clarify the harms associated with use of “spray poppers”, which are in fact a distinct group of aerosol solvent or propellant inhalants and are more dangerous than nitrite inhalant poppers–both in formulation and mode of administration (huffing) [27]. Participants in this study shared that use was common in their communities and many inaccurately considered these aerosol inhalants in the category of poppers.

The importance of neighborhood and social networks as influences on substance use in the urban gay community has been supported by previous research [52, 53]. Public health opportunities for dissemination of information about poppers exist at the intrapersonal level (e.g., websites), interpersonal level (e.g., through healthcare providers, peer educators and sexual networks) and the community level (e.g., public health campaigns focusing on neighborhoods where popper users live and socialize).

### Policy

The vague legal status of poppers (i.e., being legal to purchase but not for their intended use) potentially contributes to low perceived risk, ubiquity of use in some communities and inhibition of the provision of information about risks. This perspective, however, runs in contrast to participant lived experiences, which expressed addictive behavior and negative consequences of popper use, as well as the desire to be better informed about risks. The legal status of alcohol, cannabis and tobacco products, for example, have enabled label warnings, dissemination of health information about risks and public health campaigns aimed at reducing harms related to use.

There is also a clear need for policies and funding that support integrated and/or co-located substance use and HIV services. YMSM experience multiple barriers to accessing needed services, such as navigating complex applications for health insurance or Medicaid and having to receive care in multiple systems and locations to address their comprehensive health and wellness needs. Funding systems are often siloed and/or create barriers with restrictive eligibility requirements (e.g., the need to have a detectable HIV viral load in order to receive medical case management that can support youth with navigating systems and staying retained in care). YMSM in this study experienced high rates of unemployment, poverty and unstable housing, which is commensurate with the challenges faced by other urban YMSM [54]; it’s essential that strategies to promote substance use treatment and HIV prevention and care include support with employment and housing.

### Research

Our findings highlight several gaps and opportunities for future research, which we have prioritized below. Broadly, we recommend that any effective research on poppers should account for their use in the context of polysubstance use.

1. To more accurately understand the prevalence of popper use, including in priority subpopulations like YMSM, we advocate for improved population data and the incorporation of specific screening on popper use. Given the context for use, impact on focus populations, and distinct risk profile, we recommend that poppers be assessed beyond the broad ‘inhalant’ category. Future research should focus on developing validated screening tools on poppers for use in research and clinical settings and seeking a better understanding of the perspectives of providers serving YMSM about popper use.
2. The majority of participants in this study were introduced to poppers by a sexual partner and used poppers for the first time during a casual sexual encounter; these findings differ from other recent research indicating that poppers were commonly used among YMSM prior to sexual debut [55]. Further research is needed to learn about the context in which poppers are initiated and the temporal relationships between popper and other substance use, as a potential opportunity to develop interventions that support linkage to PrEP or other health education that could reduce risk for HIV transmission.
3. Finally, additional research is needed to understand perspectives, and potential disparities, among diverse populations. This study had a high representation of Latino participants, perhaps a result of San Diego’s location near the U.S.-Mexico border. This unplanned representation of Latino participants is unique in the popper literature, where the voices of Latinos who are highly impacted by HIV and substance use are underrepresented in research. Among YMSM (18–30 years old) diagnosed in San Diego County between 2016 and 2020, 52.5% identified as Hispanic/Latinx (S. Tweeten, County of San Diego, personal communication, May 17, 2021) and further research describing the perspectives of Latino YMSM is warranted, especially among YMSM who may be binational. This study presented the perspectives of YMSM with HIV, however perspectives of YMSM without and at-risk for HIV should also be assessed.

### Limitations

While this study offers important information about context and potential influences on popper use, it was designed to explore the lived experiences of English-speaking YMSM with HIV who use poppers in San Diego and thus may not be generalizable to other populations. As generalizability is outside of the aim of qualitative methods, clinicians should use caution when applying our findings to populations served in their setting. This sample reflected a group who was engaged in HIV care and experienced with substance use treatment. Individuals not receiving HIV care and substance use treatment may be especially vulnerable and possess unique needs and perspectives. It is also important to acknowledge that ‘saturation’ in qualitative research is a matter of degree, not a fixed point. Substance use often develops as a strategy to cope with stress and adversity; however, this study did not thoroughly assess mental health or traumatic experiences that may precede use. Information from this study was self-reported and explored stigmatized topics, potentially leading to response bias. However, we note two important aspects that would likely attenuate this potential bias: (1) the interviewer was a licensed clinical social worker with extensive experience and training working with YMSM with HIV and substance use; and (2) detailed and thoughtful participant descriptions of popper use, context of use and articulation of life experiences and sincere uncertainties surrounding use would indicate that respondents felt comfortable providing honest answers. Additionally, because all but one participant identified as gay, the extent that findings are applicable to men who identify as bisexual or have a different minority sexual orientation is not known.

This study has several strengths, especially in the new information it offers to address existing research gaps and provide context and diverse perspectives on popper use among YMSM with HIV. The inclusion of the SEM allows for an understanding of the interconnected influences on popper use, potential to intervene to reduce harms and support the health of YMSM with HIV, and suggest future research priorities. The rich data including both clear themes and diverse perspectives reinforces the study design and comfort level of participants in sharing honestly about sensitive information and lived experiences.

## Conclusions

In this sample of YMSM with HIV, poppers were frequently used during sexual encounters, often concurrently with other substances (especially methamphetamine and unprescribed erectile dysfunction medications). Participants were introduced to poppers by casual sex partners, frequently met on ‘apps’ like Grindr, and motivations for use included enhanced pleasure and intimacy, facilitation of anal sex and reduced pain during sex. Perceived risk of popper use was low; contributing factors included their legal status, short duration, contextual use (e.g., as a tool or accessory for sex), lack of health care provider assessment, and relative severity in the context of other drug use (primarily methamphetamines). Participants were not educated about the potential risks of using poppers and desired more information. Participants were engaged in both HIV care and substance use treatment and had positive, trusting relationships with their providers. Despite these supportive relationships, popper use was not addressed in either the HIV care or substance use setting. Protective factors identified included the belief that poppers could reduce tearing and bleeding during sex, support reduced use of other substances (like methamphetamine), and reinforce commitment to antiretroviral medication adherence and testing for sexually transmitted infections. Participants did not articulate a direct connection between popper use and HIV risk, but were able to identify associated risks such as impaired decision making, impacts on condom use and discussions about HIV status with sexual partners, and use of poppers associated with other risk factors such as polysubstance use and multiple partners. YMSM with HIV in this study prioritized their health through engagement in HIV care and antiretroviral medication adherence demonstrated by viral suppression. HIV care providers and substance use clinicians are trusted by YMSM and can promote their health by assessing for popper use and providing health education about related risks. Public health interventions can disseminate information at a community level through focused campaigns, which could influence social norms. Clarification of poppers’ legal status could support the availability of information about risks at point of purchase and shape YMSM’s perceptions about the risks of popper use. Future research opportunities exist to support the development of effective HIV prevention strategies among YMSM who use poppers.

Interpreting the experiences around popper use of this sample of YMSM with HIV through the lens of the SEM allows for a cohesive synthesis of the interconnectedness of each level and multiple concurrent pathways to reduce potential harm. YMSM and their partners, healthcare providers and support staff, public health entities, and policy advocates all have important roles to play. Combined, these efforts represent tangible and meaningful ways to support the health and wellbeing of YMSM with HIV.

## Data Availability

The interview guide and de-identified transcribed interviews are available upon reasonable request from the corresponding author.
